# The histone demethylase KDM5C enhances the sensitivity of acute myeloid leukemia cells to lenalidomide by stabilizing cereblon

**DOI:** 10.1186/s11658-025-00697-8

**Published:** 2025-01-29

**Authors:** Lu Zou, Dan Cao, Qing Sun, Wenjun Yu, Bingzong Li, Guoqiang Xu, Liang Zhou

**Affiliations:** 1 Jiangsu Key Laboratory of Neuropsychiatric Diseases and College of Pharmaceutical Sciences, Jiangsu Province Engineering Research Center of Precision Diagnostics and Therapeutics Development, Jiangsu Key Laboratory of Preventive and Translational Medicine for Geriatric Diseases, Suzhou Key Laboratory of Drug Research for Prevention and Treatment of Hyperlipidemic Diseases, Soochow University, 199 Ren’ai Road, Suzhou, 215123 Jiangsu China; 2 Department of Hematology, The Second Affiliated Hospital of Soochow University, San Xiang Road 1055, Suzhou, 215006 China; 3 Suzhou International Joint Laboratory for Diagnosis and Treatment of Brain Diseases, College of Pharmaceutical Sciences, Soochow University, Suzhou, 215123 Jiangsu China; 4 MOE Key Laboratory of Geriatric Diseases and Immunology, Suzhou Medical College of Soochow University, Suzhou, 215123 Jiangsu China

**Keywords:** Cereblon (CRBN), KDM5C, Leukemia, Lenalidomide, Cell viability

## Abstract

**Background:**

The protein cereblon (CRBN) mediates the antileukemia effect of lenalidomide (Len). Len binds to CRBN, recruits IKZF1/IKZF3, and promotes their ubiquitination and degradation, through which Len exhibits its antileukemia and antimyeloma activity. Therefore, the protein level of CRBN might affect the antiproliferative effect of Len. In this study, we explored the interactome for CRBN using proximity labeling technique TurboID and quantitative proteomics, and then investigated the antileukemia effect of Len.

**Methods:**

The primary acute myeloid leukemia (AML) cells and AML cell lines were used to explore the functions of histone demethylase KDM5C on the antileukemia effect of Len. The cell viability and CRBN protein levels were evaluated in these cell lines. In addition, the KDM5C inhibitors were used to determine the effects of KDM5C enzymatic activity on the viability of AML cell lines.

**Results:**

We identified that histone demethylase KDM5C was a CRBN-interacting protein. Biochemical experiments found that the CRBN-interacting protein KDM5C could stabilize CRBN and enhance the antileukemia effect of Len in an enzyme activity-independent manner. Furthermore, our studies revealed that the small-molecule compound MLN4924 could increase CRBN by elevating KDM5C.The combination of MLN4924 and Len can further increase the sensitivity of primary AML cells and AML cell lines to Len.

**Conclusions:**

This study provides a possible strategy for a combination treatment with MLN4924 and Len for leukemia.

**Supplementary Information:**

The online version contains supplementary material available at 10.1186/s11658-025-00697-8.

## Background

Leukemia and multiple myeloma (MM) are blood cell cancer [1, 2].Clinical drugs, such as the proteasome inhibitor bortezomib, immunomodulatory drugs (IMiDs), lenalidomide (Len) and pomalidomide (Pom), have been approved by the FDA for the treatment of blood cancer [3–5].Due to the lack of early diagnosis and drug resistance, these diseases progress rapidly and become fatal. Therefore, novel methods and strategies for the diagnosis and treatment of leukemia and MM are needed.

Cullin 4-RING E3 ligase (CRL4^CRBN^) consists of cullin 4A/B, DDB1, ROC1, and CRBN [6–8]. As a substrate receptor of the CRL4 E3 ligase, CRBN binds to different substrates and promotes the ubiquitination of these substrates. Furthermore, CRBN is the primary target of thalidomide (Thal) and its analogs Len and Pom [9]. Therefore, CRBN mediates the teratogenic effect of Thal [9] and the anticancer effects of Len and Pom in blood cancer [7, 8].

Upon binding to Len, CRBN can recruit and promote the ubiquitination-mediated degradation of new substrates (neo-substrates) of IKZF1/3 in AML cells [10]. Therefore, the sensitivity of AML to Len could be affected by the protein level of CRBN [11]. In line with this, CRBN deficiency in cells leads to Len and Pom resistance [12, 13]. The anticancer effect of Len could be enhanced by arsenic trioxide through increasing *CRBN* mRNA levels [14]. The attenuation of the ubiquitination-mediated degradation of CRBN also enhances the anticancer effect of Len [15, 16]. The blockage of CRBN cleavage stabilized CRBN and then potentiated the anticancer effect of Len [17, 18]. The small-molecule inhibitor SHIN1 increased CRBN by blocking the autophagic degradation of CRBN and then enhanced the anticancer effect of Len [19]. Taken together, these data demonstrated that CRBN modulated Len sensitivity.

Here, we identified the histone demethylase KDM5C in the CRBN interactome. Our proteomic and biochemical approaches revealed that KDM5Ccould bind to and stabilize CRBN, and the KDM5C inhibitor could not affect CRBN, which suggest that KDM5C regulated CRBN in an enzyme activity-independent manner. Furthermore, we discovered that the small-molecule compound MLN4924 increased KDM5C and subsequently stabilized CRBN, therefore potentiating the antileukemic effect of Len. Our discovery revealed that the histone demethylase KDM5C regulated the sensitivity of AML to Len, which might benefit the combination therapy for leukemia patients.

## Methods

### Reagents

The small-molecule compounds and reagents used in this work were purchased from the following companies: anacardic acid (AA, HY-N2020), bafilomycin A1 (Baf A1, HY-100558), 2-bromohexadecanoic acid (2-BP, HY-111770), cycloheximide (CHX, HY-12320), and Y-27632 (HY-10071) were obtained from MedChemExpress. Cell Counting Kit-8 (CCK-8, B34304), MLN4924 (S7109), KDM5-IN-1 (HY-100014), CPI-455 (HY-100421), lenalidomide (Len, CC-5013), pomalidomide (Pom, CC-4047), and MG132 (S2619) were ordered from Selleck. Biotin (V900418) was acquired from Sigma–Aldrich. NeutrAvidin agarose resin (29,201) was purchased from Thermo Fisher Scientific. Protein A/G agarose resin (36403ES08) was obtained from Yeasen Biotechnology (Shanghai, China). Anti-HA magnetic beads (B26201), FLAG peptide (B23111), and FLAG affinity gel (B23102) were acquired from Bimake.

The following antibodies were purchased from the following companies: anti-FLAG M2 (F1084) from Sigma; anti-KDM5C (ab194288) from Abcam; anti-CRBN (D8H3S),anti-H3K4me3 (9751 s),and anti-H3 (4499 s) were obtained from Cell Signaling Technology; anti-HA (51,064–2-AP), anti-β-actin (20,536–1-AP), and anti-GAPDH (60,004–1-IG) were ordered from ProteintechGroup; anti-GFP (CPA9056), anti-HA (CPA9058), and anti-IKZF1 (MB0092) were acquired from Bioworld. The secondary antibodies (111–035-045 and 115–035-062) were obtained from Jackson ImmunoResearch.

### Plasmid construction

Plasmids were constructed according to a previous procedure [20, 21]. The sh*RNA* plasmids were generated using the pLKO.1-TRC lentiviral vector according to a previous method [22]. KDM5C was obtained from MiaoLing Biology. The FLAG-KDM5C forward oligonucleotide (5′-GAGGTGACCCTGGATGAGAA-3′), KDM5C reverse complementary oligonucleotide (5′-CAGGAGCTGAGGTCTGAAC-3′), CRBN forward oligonucleotide (5′-ATGCTGAGACCTTAATGGACAGA-reverse-3′), and CRBN reverse oligonucleotide (5′-AAGTCGCTGGATAGCACTGC-3′) were synthesized by GeneWiz (China). All constructed plasmids were verified by DNA sequencing (GeneWiz).

### Primary AML cells and cell lines

The human embryonic kidney cell line HEK293T, cervical cancer cell line HeLa, mouse hippocampal neuronal cell line HT22, and AML cell lines NB4, K562 and THP-1 were obtained from the American Type Culture Collection (ATCC). MV4-11 was a kind gift from Dr. Dong Chen at Soochow University. NB4, K562, THP-1, and MV4-11 cells were cultured in basic RPMI 1640 medium (C11875500BT, Gibco). HEK293T, HT22 and HeLa cells were cultured in Dulbecco’s modified Eagle’s medium (DMEM, SH30243.01, HyClone). The growth media were supplemented with 10% fetal bovine serum (FBS, YS210414, EallBio Life Sciences), 100 units/mL penicillin and 100 µg/mL streptomycin (C100C5, NCM Biotech). The primary AML cells were separated from the bone marrow sample. Cells were then cultured in RPMI-1640 medium. The use of human tissue samples was approved by the Ethics Committee of the Second Affiliated Hospital of Soochow University (Approval No: SUDA20240522H04, date 1 November 2024).

### Construction of stable cell lines

The sh*RNA*-EGFP vector with short hairpin RNA (shRNA) sequence targeting the KDM5C gene (GAGAGGAGCTAGAGCCAAA) was purchased from GeneChem (GENE: REVG006-1). A lentiviral sh*RNA* vector was used as a control (Ctrl-sh*NC*). Both vectors contained enhanced green fluorescence protein (EGFP) and a puromycin resistance marker. Lentiviral particles were generated according to a previously described method [23]. Briefly, the sh*NC* or sh*KDM5C* lentiviral particles were transfected into HEK293T, K562, and NB4 cell lines, which were subsequently selected with 1 µg/mL puromycin (P8230, Solarbio Life Sciences) for two weeks. Immunofluorescence and immunoblotting were used to verify the knockdown efficiency of KDM5C in HEK293T, K562 and, NB4 cells.

### si*RNA* and plasmid transfection

si*RNAs* were synthesized by Guangzhou RiboBio Co (China). HEK293T cells were transfected with si*NC* (Cat #: 160,818), si*KDM5C* #1(target sequence: GAGAGGAGCUAGAGCCAAA), si*KDM5C #2*(target sequence: CACACUUGAGGCCAUAAUC), and si*KDM5C #3* (target sequence: CAGAGAAGCUAGACCUGAA) using LipoRNAiMAX (13,778–150, Invitrogen). The plasmid of pLVX-FLAG-KDM5C was transfected using Lipofectamine 3000 (L3000-015, Invitrogen).

### Proximity-labeling and MS analysis

The optimized proximity-dependent biotinylation assay was performed according to our previous study [24]. Briefly, the plasmids of pcDNA3.1, CRBN-FLAG-NLS-TurboID, and CRBN-FLAG-NES-TurboID were transfected into HEK293T cells for 48 h, and subsequently treated with biotin (50 μM) for 30 min. The HEK293T cells were collected, lysed, and the resulting cell lysates were digested for MS analysis using a procedure described previously [24].

### Quantitative PCR (qPCR)

The qPCR was performed according to a previous method [25]. Briefly, total RNA was isolated using TRIzol (R401-01, Vazyme), and then HiScript III All-in-one RT SuperMix (R333, Vazyme) was used to synthesize the cDNA library. A Biotool SYBR Green One Step qRT‒PCR Kit was used to perform qPCR on an Applied Biosystems 7500 Real‒Time PCR system. All the results were normalized to that of *GAPDH*. The qPCR primers (*KDM5C*-forward: GGGTCCGACGATTTCCTACC; *KDM5C*-reverse: ATGCCCGATTTCTCTGCGATG; *CRBN* forward: ATGCTGAGACCTTAATGGACAGA; *CRBN* reverse: AAGTCGCTGGATAGCACTGC; *β-actin* forward: GGGAAATCGTGCGTGACATT; and *β-actin* reverse: GGAACCGCTCATTGCCAAT) were synthesized and purified via HPLC by GeneWiz.

### CCK-8 assay

The relative cell viability was measured using a CCK-8 assay. Cells were seeded and cultured in 96-well plates (1000 cells/well) for different durations after drug treatment. CCK-8 reagent (10 µL/well) was added to the 96-well plates and incubated for 1 h at 37 °C. The absorbance was measured at 450 nm by a Tecan Infinite M1000 PRO (Switzerland).

### Protein stability experiments

Cycloheximide (CHX) chase experiments were performed to measure protein degradation. HEK293T cells overexpressing FLAG or FLAG-KDM5C were treated with CHX (100 µg/mL) for the indicated times. For proteasome inhibition experiments, HEK293T cells stably expressing si*NC* or si*KDM5C* were treated with DMSO or MG132 (10 µM) for 12 h and the lysosomal inhibitor Baf A1 (200 nM) for 24 h. The cell lysates were harvested and subjected to immunoblotting analysis.

### Western blotting analysis

Cell lysates or immunoprecipitates were harvested and subjected to western blotting according to a previously described method [26]. NcmECL Ultra substrate (P10300, NCM Biotech) was used to visualize and analyze protein expression.

### lmmunoprecipitation

FLAG affinity gel (B23102, Bimake) was used to purify FLAG-tag proteins. Cells expressing FLAG-tag proteins were lysed in RIPA buffer supplemented with protease inhibitor cocktail (B14012, Bimake). After removal of cell debris by centrifugation, 50µL FLAG affinity gel was added to 500 µg of protein extract and incubated overnight at 4 °C. The beads were then washed three times and boiled with 2 × SDS loading buffer for western blotting. The endogenous immunoprecipitation assays were performed with the 1 µg IgG/KDM5C antibody and 50µL protein A/G agarose beads. Specific antibodies against FLAG M2 (1:1,0000, F1084, Sigma), HA (1:1,000,51,064–2-AP, Proteintech Group), KDM5C (1:1,000, ab194288, Abcam), CRBN(1:1,000, 71,810, CST), GAPDH(1:l,0000, 60,004–1-IG, Proteintech Group), and the secondary antibodies (111–035-045 and 115–035-062, Jackson ImmunoResearch) were used to detect the indicated proteins.

## Results

### The histone demethylase KDM5C interacts with CRBN

**Fig. 1:**
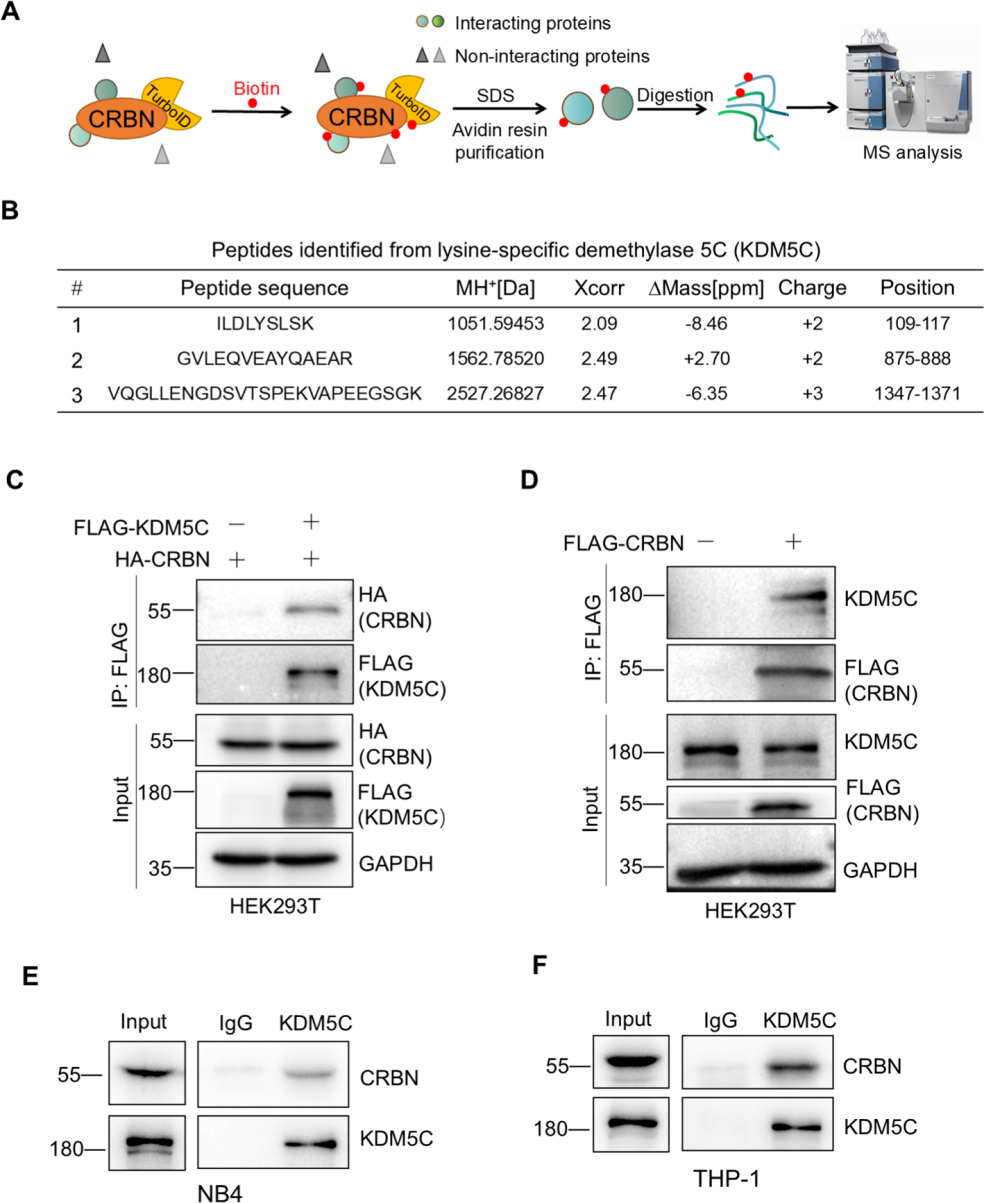
The histone demethylase KDM5C interacts with CRBN. **A** The CRBN-interacting proteins were labeled with biotin, purified with NeutrAvidin, digested with trypsin, and analyzed by LC‒MS/MS. **B** Information for MS-identified tryptic peptides from KDM5C. **C** HA-CRBN interacted with FLAG-KDM5C. HEK293T cells were transfected with the indicated plasmids for 48 h, collected, lysed, and subjected to immunoprecipitation assay and western blotting. **D** Exogenous CRBN interacted with endogenous KDM5C. The plasmids of pcDNA3.1 or FLAG-CRBN were transfected into HEK293T cells for 48 h. The cells were collected, lysed, and subjected to immunoprecipitation and western blotting. **E**, **F** Endogenous KDM5C interacted with endogenous CRBN in AML cells. NB4 (**E**) or THP-1 (**F**) cells were collected, lysed, and subjected to immunoprecipitation and western blotting using the indicated antibodies

To discover new regulators for CRBN, we used the proximity labeling technique TurboID and quantitative proteomics to identify the CRBN-interacting proteins (Fig. 1A). The experiments identified NAC1, PICALM, and histone demethylase KDM5C as specific CRBN-interacting proteins (Fig. 1B and Supplemental Fig. 1A). To further confirm these results from mass spectrometry, we transfected the HA-CRBN and FLAG-KDM5C, FLAG-PICALM, or FLAG-NAC1 plasmids into HEK293T cells and then examined the interaction between CRBN and KDM5C, PICALM, or NAC1. Coimmunoprecipitation assays demonstrated that Flag-KDM5C, FLAG-PICALM, or FLAG-NAC1 bound to HA-CRBN (Fig. 1C and Supplemental Fig. 1B). We also used Flag-CRBN to precipitate endogenous KDM5C, and the results revealed that CRBN could bind to endogenous KDM5C (Fig. 1D). Furthermore, we performed the endogenous coimmunoprecipitation assays in two AML cell lines THP1 and NB4, and the results disclosed that endogenous KDM5C could bind to endogenous CRBN in AML cell lines (Fig. 1E, F). Taken together, these data suggested that the histone demethylase KDM5C interacted with CRBN.

### KDM5C stabilizes CRBN

**Fig. 2:**
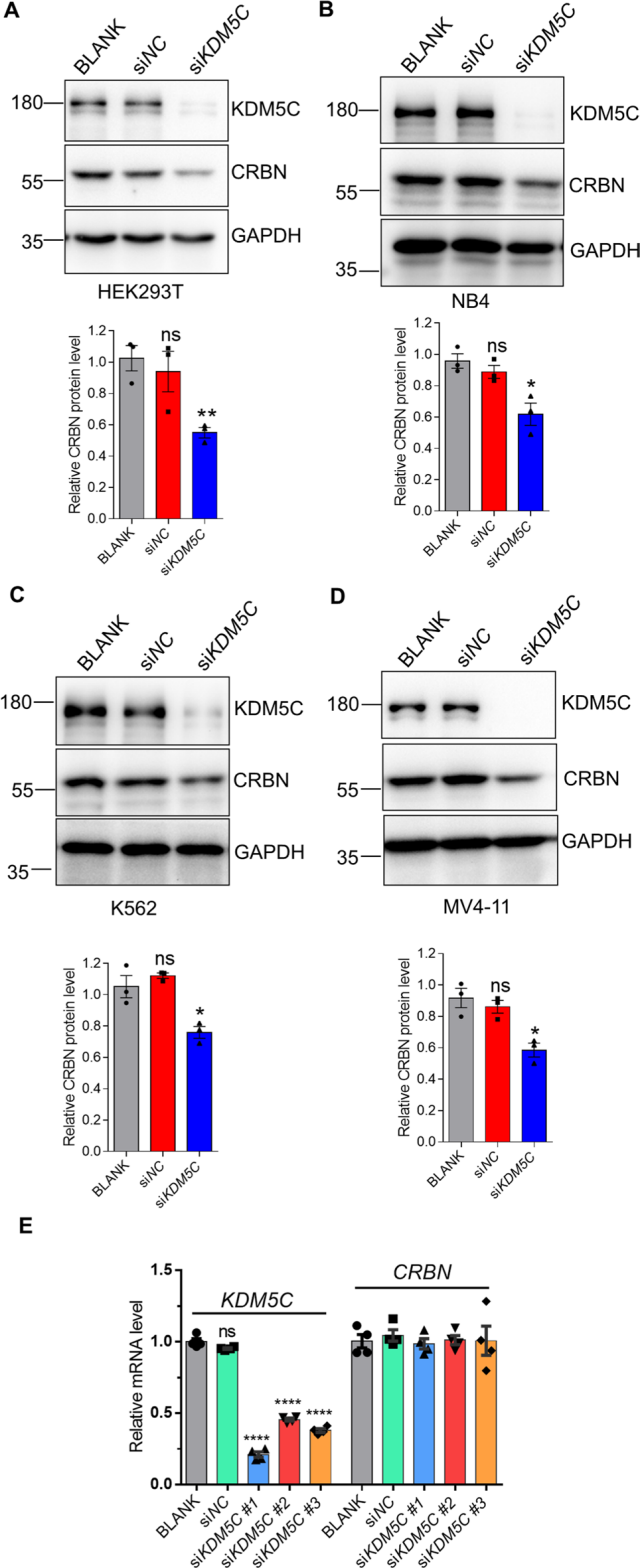
KDM5C stabilizes CRBN. **A** KDM5C and CRBN were analyzed by western blotting after KDM5C was knocked down by si*RNA* in HEK293T cells for 48 h. **B**–**D** Western blotting was used to analyze KDM5C and CRBN in NB4 (**B**), K562 (**C**), and MV4-11 cells (**D**). The cells were transfected with si*RNA* for 48 h, collected, lysed, and subjected to western blotting using the indicated antibodies. **E** CRBN mRNA levels were analyzed by qPCR. The si*KDM5Cs* were transfected into HEK293T cells, and the mRNAs were extracted for qPCR analysis. The data are shown as the means ± SEMs (n ≥ 3). Student’s *t* test, *P < 0.05; **P < 0.01; ****P < 0.0001; ns: not significant

Our proteomic and biochemical methods demonstrated that KDM5C interacted with CRBN (Fig. 1). As a histone demethylase, KDM5C regulates gene transcription by removing methyl groups from lysine 4 of histone H3 in an enzyme activity-dependent manner [27]. We then sought to examine the possible regulatory effect of KDM5C on CRBN. We overexpressed KDM5C in HEK293T cells and then immunoblotted the cell lysates. The results indicated that KDM5Cstabilized CRBN, as the protein level of CRBN increased in the KDM5C-overexpressing HEK293T cells (Supplemental Fig. 2). Conversely, we also knocked down endogenous KDM5C in HEK293T cells. CRBN was decreased in KDM5C-deficient HEK293T cells (Fig. 2A). CRBN mediated the anticancer effects of lenalidomide. We then validated the regulatory effect of KDM5C on CRBN in the AML cell lines NB4, K562, and MV4-11. The results were consistent with the findings in HEK293T cells that KDM5C stabilized CRBN in leukemia cells (Fig. 2B–D). Furthermore, the qPCR results indicated that KDM5C did not alter the mRNA level of CRBN (Fig. 2E). Taken together, these results suggested that KDM5C increased CRBN at the protein level. Since KDM5C is a demethylase that can remove lysine tri-/dimethyl modifications from histone H3 and nonhistone substrates, we investigated whether demethylase activity is required for the regulation of KDM5C on CRBN protein levels. We used two traditional KDM5 inhibitors, CPI-455 and KDM5-IN-1, to suppress the activity of the demethylase KDM5C and then examined the protein level of CRBN. The western blotting results indicated that blocking KDM5C indeed increased H3K4me3 but did not affect the protein levels of CRBN (Supplemental Fig. 3), which suggested that the enzyme activity of KDM5C was not essential for the regulation of CRBN by KDM5C.

### The small-molecule compound MLN4924 increases KDM5C and CRBN

**Fig. 3:**
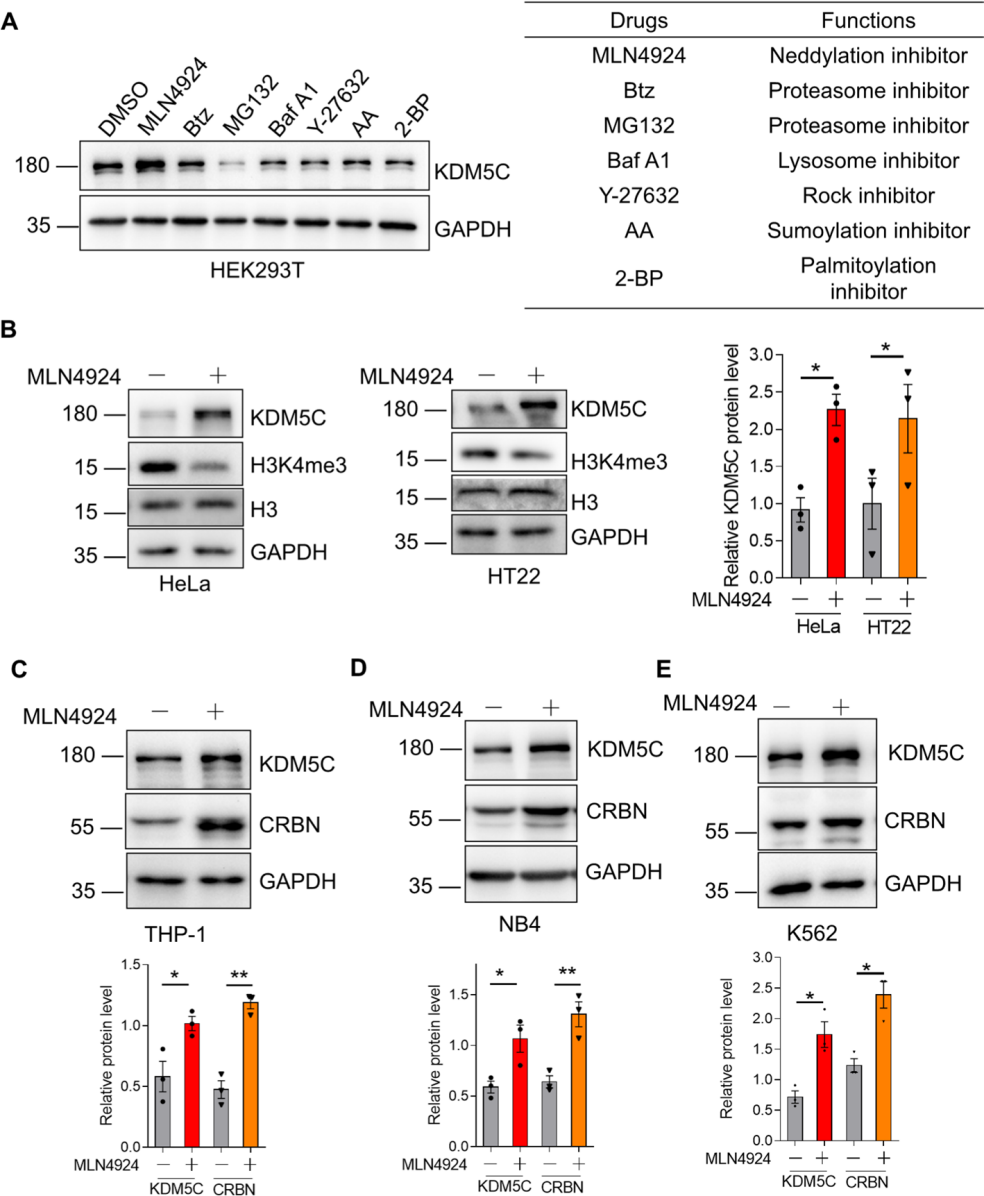
The small-molecule compound MLN4924 increases KDM5C and CRBN. **A** HEK293T cellswere treated with various types of inhibitors (1 µM MLN4924, 1 µM Btz, 1 µM MG132, 100 nM Baf A1, 1 µM Y-27632, 1 µM AA, and 1 µM 2-BP) for 24 h and the resulting cell lysates were subjected to western blotting. **B**–**E** MLN4924 (1 µM) was used to treat HeLa, HT22 (**B**), THP-1 (**C**), NB4 (**D**), and K562 (**E**) cells for 24 h and the cell lysates were used for western blotting. The data are shown as the means ± SEMs (*n =* 3).Student’s *t*test, *P < 0.05; **P < 0.01; ns: not significant

To further investigate the possible degradation pathways by which KDM5C regulates CRBN, we blocked the ubiquitin‒proteasome system (UPS) and the autophagy‒lysosome pathway (ALP) using their inhibitors MG-132 and bafilomycin A1. The results revealed that blocking the UPS and ALP did not restore the protein levels of CRBN in *KDM5C*-deficient cells (Supplemental Fig. 4), which indicated that the two major cellular degradation pathways were not required for the regulation of CRBN by KDM5C. CRBN mediated the antiproliferative effects of Len in blood cancer cells. To identify the possible small-molecule compounds that increase KDM5C and therefore enhance the anticancer effects of Len in leukemia. We treated HEK293T cells with inhibitors for different signaling pathways. The results demonstrated that the neddylation inhibitor MLN4924 increased KDM5C (Fig. 3A). We further confirmed this finding using two different cell lines, HeLa and HT22. MLN4924 increased KDM5C and CRBN in these two cell lines (Fig. 3B). Immunoblotting of the KDM5C substrate H3K4me3 unveiled that H3K4me3 decreased upon MLN4924 treatment (Fig. 3B). To further confirm the regulatory effect of KDM5C on CRBN in AML cell lines, we treated THP-1, NB4, and K562 cells with MLN4924.These results were consistent with the data from nonleukemia cell lines showing that MLN4924 increased KDM5C and CRBN in AML cell lines (Fig. 3C–E).

### MLN4924 stabilizes CRBN by increasing KDM5C

**Fig. 4:**
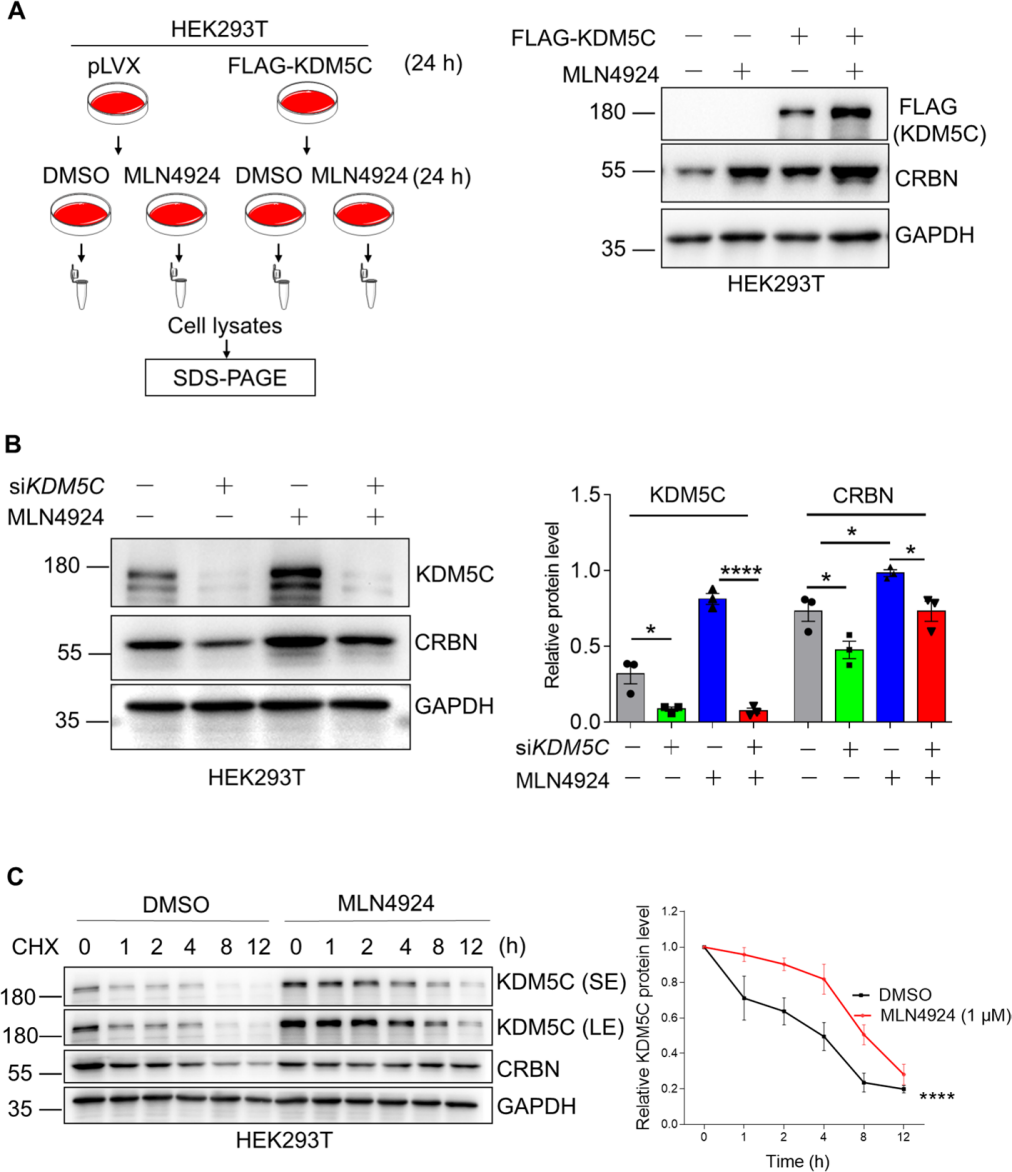
MLN4924 stabilizes CRBN by increasing KDM5C. **A** HEK293T cells were transfected with the FLAG-KDM5C plasmid for 24 hand treated with MLN4924 (1 µM) for 24 h, and whole-cell lysates were immunoblotted for KDM5C. **B** MLN4924 did not increase CRBN in KDM5C deficient HEK293T cells. HEK293Tcells were transfected with si*KDM5C*for 48 h, treated with MLN4924 (1 µM) for 24 h, and lysed for Western blotting. **C** HEK293T cells were treated with 1 µM MLN4924 for 12 h, and then the cells were treated with the 100 µg/mL CHX for the indicated time. The data are shown as the means ± SEMs (*n =* 3), Student’s *t* test,*P < 0.05;****P < 0.0001

The aforementioned experiments demonstrated that MLN4924 increased KDM5C and CRBN (Fig. 3).We next sought to validate the essential role of KDM5C in the regulation of CRBN by MLN4924. To do so, we overexpressed KDM5C in HEK293T cells and then treated the cells with MLN4924. Immunoblotting of cell lysates revealed that MLN4924 could increase exogenous KDM5C and further stabilize CRBN (Fig. 4A). These data suggested that MLN4924 could stabilize CRBN by increasing KDM5C. To further demonstrate that MLN4924 regulated CRBN through KDM5C, we knocked down KDM5C in HEK293T cells using si*KDM5C* and then treated these cells with MLN4924. We disclosed that MLN4924 did not increase CRBN in *KDM5C*-deficient cells (Fig. 4B), which suggested that MLN4924 increased CRBN by stabilizing KDM5C. Consistent with this finding, our CHX-chase experiments further revealed that MLN4924 stabilized KDM5C and CRBN (Fig. 4C).

### MLN4924 enhances the antileukemia effect of Len

**Fig. 5:**
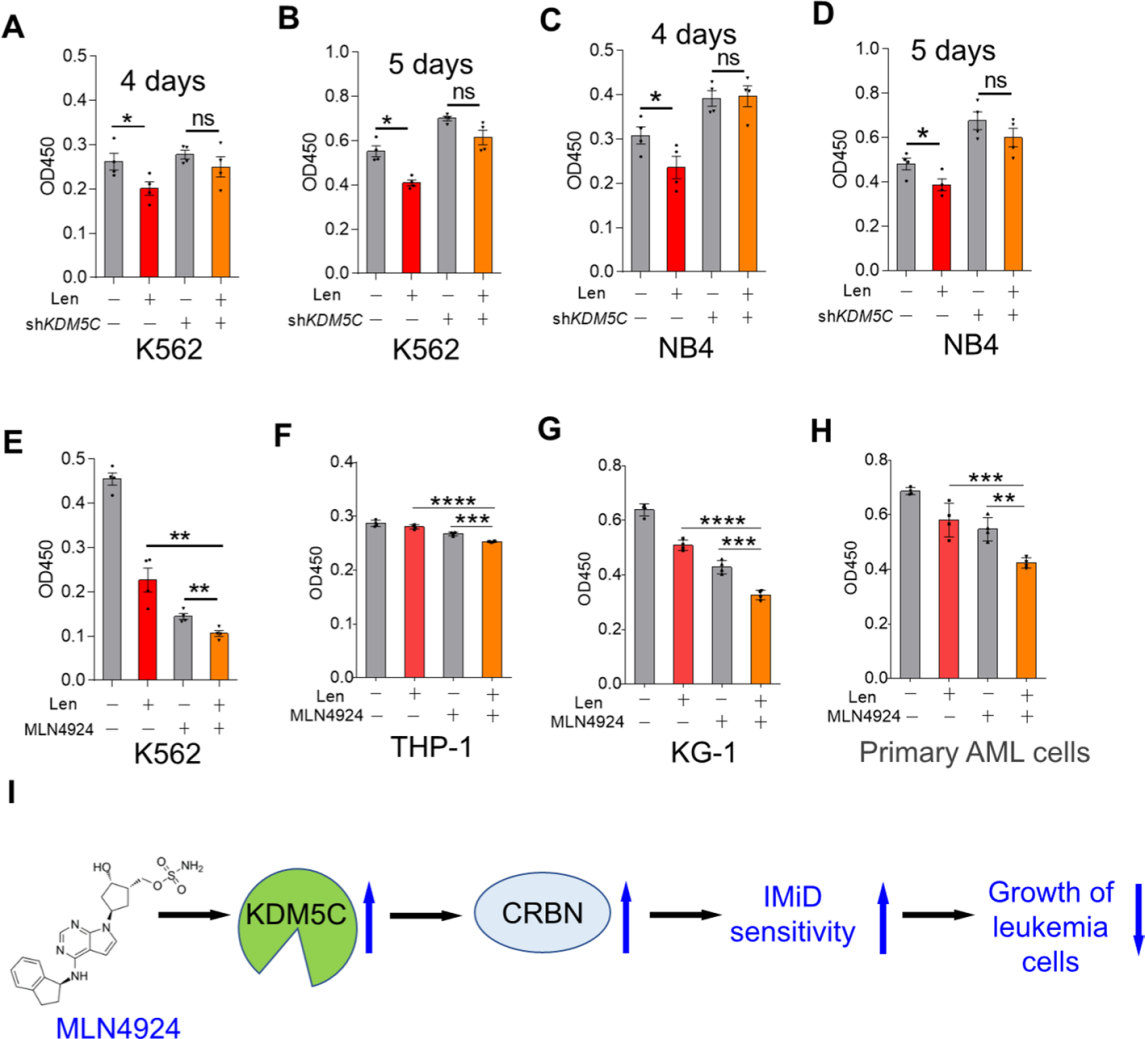
MLN4924 enhances the antileukemia effect of Len. **A**–**D** K562 (**A**, **B**) and NB4 (**C**, **D**) cells with stable *KDM5C* knockdown were treated with lenalidomide (10 µM) for 4 (**A** and **C**) or 5 (**B** and **D**) days, and the cell proliferation was examined by CCK-8. sh*NC* was used as a negative control. The data are shown as the means ± SEMs (*n =* 4), and Student’s* t* test was used to calculate the *P* values. *P < 0.05; ns: not significant. **E–H** K562, THP-1, KG-1, and primary AML cells were treated with MLN4924 (40 nM) for 18 h, and then treated with lenalidomide (10 µM) for 4 days. Cell viability was measured by a CCK-8 assay. The data are shown as the means ± SEMs (*n =* 4); **P < 0.01;***P < 0.001;****P < 0.0001; ns: not significant. **I** Proposed model for the regulation of KDM5C on the antileukemia effect of IMiDs

As KDM5C stabilized CRBN, we hypothesize that the antileukemia effects should be attenuated in KDM5C-deficient cells. We treated stable sh*NC* and sh*KDM5C* AML cell lines K562 and NB4 with Len, and the results indicated that Len did not inhibit the proliferation of stable sh*KDM5C* AML cell lines K562 and NB4 (Fig. 5A–D). Given that MLN4924 increased CRBN through KDM5C, which mediates the antimyeloma effects of Len, we further explored whether MLN4924 could regulate the antileukemia activity of Len. To this end, we treated the primary AML cells and three different AML cell lines, K562, THP-1, and KG-1, with MLN4924 and/or Len, and then assessed the cell proliferation using CCK-8 assays. Our results indicated that MLN4924 enhanced the antileukemic effects of Len (Fig. 5E–H and Supplemental Fig. 5).We also used the Len analog Pom to treat K562 and KG-1 cells, and the results disclosed that MLN4924 potentiated the antileukemia effect of Pom (Supplemental Fig. 6). Furthermore, biochemical experiments demonstrated that MLN4924 further decreased the protein level of IKZF1 upon Len treatment (Supplemental Fig. 7). Taken together, our data suggested that MLN4924 enhanced the antileukemic effect of IMiDs in primary AML cells and AML cell lines (Fig. 5I).

## Discussion

Mutations in KDM5C and CRBN resulted in intellectual disability [28–30], which suggested a possible correlation between KDM5C and CRBN. Here, we demonstrated that KDM5C could potentiate the antileukemia effect of Len through stabilizing CRBN. However, the possible functions of KDM5C and CRBN in intellectual disability need to be further investigated. Our results indicated that the enzyme activity of KDM5C was not essential for the regulation of CRBN by KDM5C (Supplemental Figs. 1, 2), which suggested that the nonenzymatic functions of KDM5C are involved in the sensitivity of AML to IMiDs. Furthermore, we revealed that the small molecule compound MLN4924 increased KMD5C and subsequently stabilized CRBN, therefore potentiating the antileukemia effect of IMiDs and providing a novel molecular mechanism for the treatment of AML using MLN4924 and IMiDs.

CRBN can be degraded through the UPS [31, 32] and ALP [19]. However, we found that UPS and ALP were not required for the regulation of KDM5C on CRBN. Our previous study showed that CRBN can be cleaved upon caspase-8 activation [17]. However, we did not observe the cleaved bands of CRBN, which indicated that caspase-8 might not be required for the regulation of KDM5C on CRBN. Therefore, the molecular mechanisms underlying the regulatory effect of KDM5C on CRBN need to be further investigated.

Methylation is a well-known epigenetic event in AML [33]. Histone and DNA methylations are deposited and eliminated by the corresponding epigenetic enzymes, and could control gene regulation in AML. Here, we revealed a novel role for the histone demethylase KDM5C in the enhancement of the antileukemia effect of IMiDs. Although our results revealed that the enzyme activity of KDM5C was not required for this regulation, epigenetic modifiers, such as the DNA methyltransferase DNMT3A [34] and histone methyltransferase EZH2 [35, 36], could regulate the disease stages of AML. Therefore, the roles of epigenetic enzymes in AML are complicated and waiting to be solved.

## Conclusions

This work revealed that the histone demethylase KDM5C regulated the protein stability of CRBN by its non-enzymatic function and that the MLN4924-KDM5C axis increased CRBN, thus potentiating the antileukemia effect of Len, which may provide an alternative combination therapy for AML.

## Supplementary Information

Additional file 1.

## Data Availability

The raw MS data are available on the ProteomeXchange Consortium (http://proteomecentral.proteomexchange.org) via the iProx partner repository with the dataset identifier PXD045100 (https://proteomecentral.proteomexchange.org/cgi/GetDataset?ID=PXD045100 or https://www.iprox.cn//page/project.html?id=IPX0007056000).

## Abbreviations

CRBN: Cereblon
Len: Lenalidomide
Pom: Pomalidomide
IMiDs: Immunomodulatory drugs
AML: Acute myeloid leukemia
UPS: Ubiquitin‒proteasome system
ALP: Autophagy‒lysosome pathway
shRNA: Short hairpin RNA
CHX: Cycloheximide
AA: Anacardic acid
2-BP: 2-Bromohexadecanoic acid
