# Supplementary material for: The histone demethylase KDM5C enhances the sensitivity of acute myeloid leukemia cells to lenalidomide by stabilizing cereblon
Source: Cell Mol Biol Lett. 2025 Jan 29;30:14. doi: 10.1186/s11658-025-00697-8 (PMC11780777; doi:10.1186/s11658-025-00697-8)
Supplement: Supplementary file 1 — Additional file 1. [file 11658_2025_697_MOESM1_ESM.pdf]

Supplementary Material

for

The histone demethylase KDM5C enhances the sensitivity of acute myeloid leukemia cells to lenalidomide by stabilizing cereblon

Lu Zou[1], Dan Cao[1], Qing Sun[1], Wenjun Yu[1], Bingzong Li[2], Guoqiang Xu[1,3,4*], Liang Zhou[1*]

[1]Jiangsu Key Laboratory of Neuropsychiatric Diseases and College of Pharmaceutical Sciences, Jiangsu Province Engineering Research Center of Precision Diagnostics and Therapeutics Development, Jiangsu Key Laboratory of Preventive and Translational Medicine for Geriatric Diseases, Suzhou Key Laboratory of Drug Research for Prevention and Treatment of Hyperlipidemic Diseases, Soochow University, 199 Ren'ai Road, Suzhou, Jiangsu 215123, China.

[2]Department of Hematology, The Second Affiliated Hospital of Soochow University, San Xiang Road 1055, Suzhou, 215006, China.

[3]Suzhou International Joint Laboratory for Diagnosis and Treatment of Brain Diseases, College of Pharmaceutical Sciences, Soochow University, Suzhou, Jiangsu 215123, China.

[4]MOE Key Laboratory of Geriatric Diseases and Immunology, Suzhou Medical College of Soochow University, Suzhou, Jiangsu Province 215123, China.

A

Peptides identified from phosphatidylinositol binding clathrin assembly protein (PICALM)

#	Peptide sequence	MH^{+}[Da]	Xcorr	ΔMass[ppm]	Charge
1	ITAAQHSVTGSAVSK	1456.77458	3.57	-0.62	2
2	ATTLSNAVSSLASTGLSLTK	1922.04546	3.16	0.68	2
3	QAALEEEQAR	1144.55364	2.8	-4.99	2
4	SGLQGYDMSTFIR	1474.68901	1.95	-7.15	2

Peptides identified from nucleus accumbens-associated protein 1 (NAC1)

#	Peptide sequence	MH+[Da]	Xcorr	ΔMass[ppm]	Charge
1	VEALPEQVAPESR	1424.73828	2.74	0.21	2
2	LLASFFDR	968.5192	2.03	-0.81	2

B

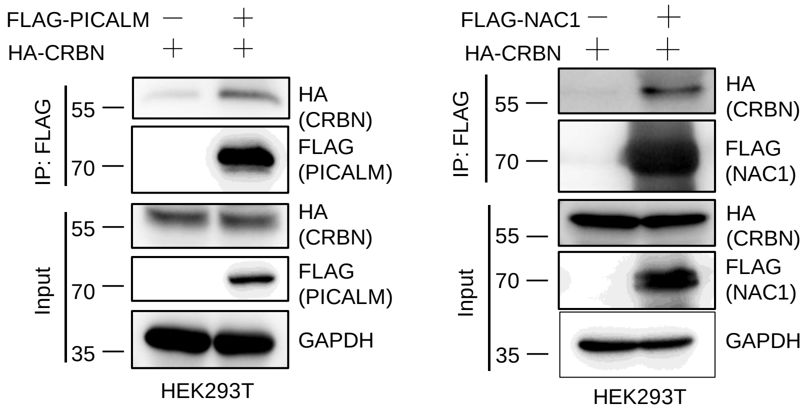

Supplemental Figure 1. PICALM and NAC1 interact with CRBN. A. Information for MS-identified tryptic peptides from PICALM and NAC1. **B.** Coimmunoprecipitation demonstrated that CRBN interacted with PICALM and NAC1 in HEK239T cells. The HEK293T cells were transfected with the indicated plasmids for 48 h, collected, lysed, and subjected to immunoprecipitation and immunoblotting.

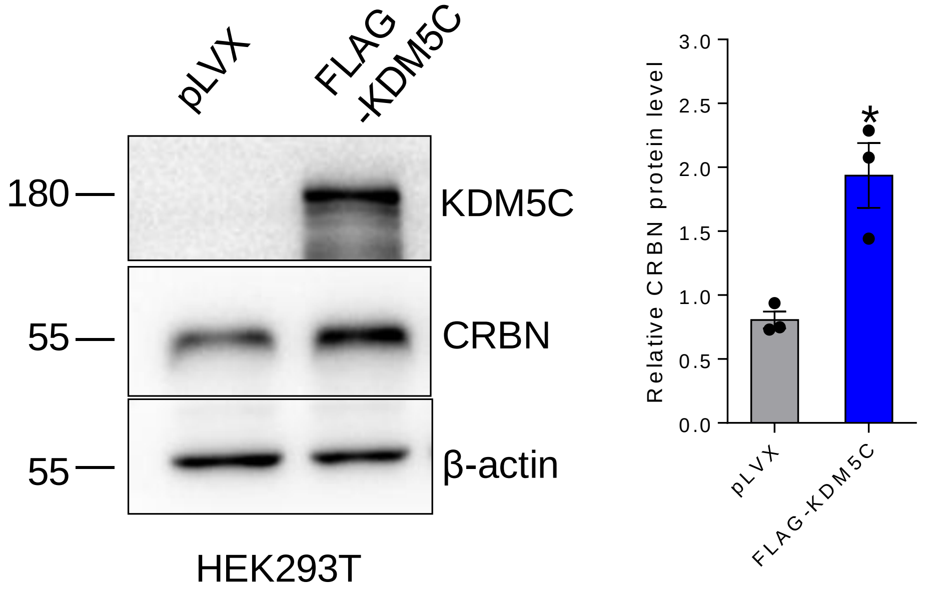

Supplemental Figure 2. KDM5C stabilizes CRBN in HEK293T. FLAG-KDM5C was overexpressed in HEK293T cells for 48 h, and KDM5C and CRBN were analyzed by Western blotting. The data are shown as the means±SEMs (n = 3). Student's *t* test, *: $P < 0.05$.

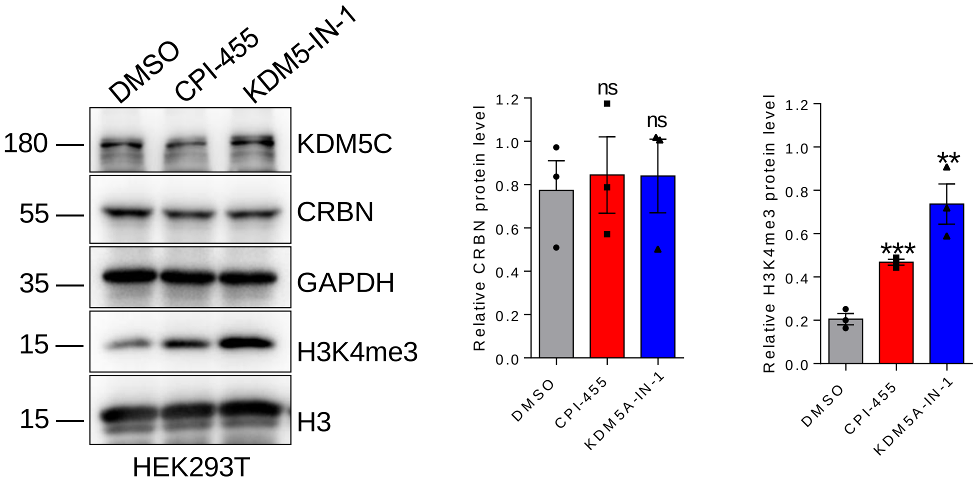

Supplemental Figure 3. KDM5C inhibitors do not regulate CRBN in HEK293T. HEK293T cells were treated with the KDM5C inhibitors CPI-455 (1 μM) and KDM5-IN-1 (1 μM) for 24 h. The resulting cell lysates were subjected to immunoblotting analysis using the indicated antibodies. Means±SEMs (n = 3), Student's *t* test, **: $P < 0.01$; ***: $P < 0.001$; ns: not significant.

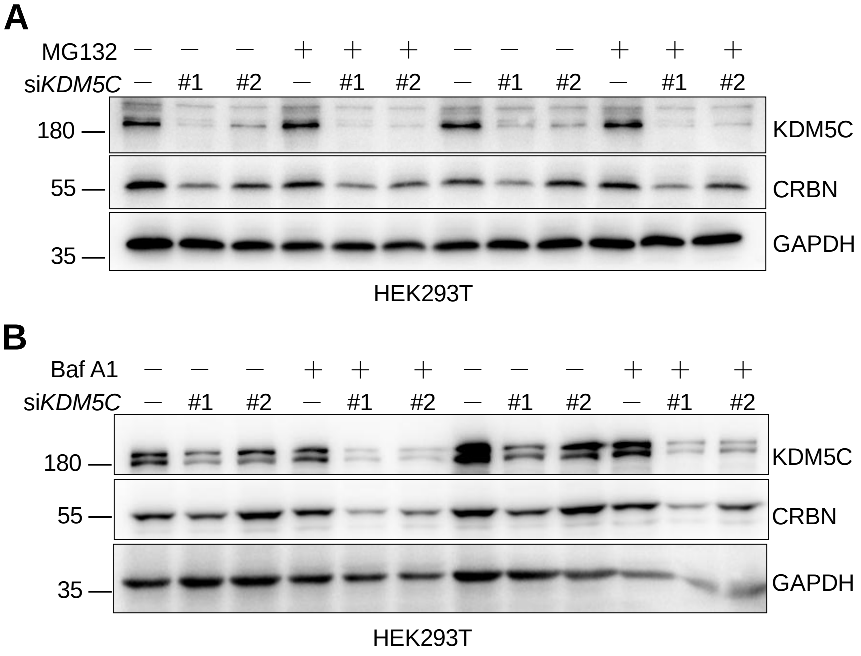

Supplemental Figure 4. The proteasome and lysosome are not required for the regulation of CRBN by KDM5C.

A-B. HEK293T cells were treated with the proteasome inhibitor MG132 (10 μM) for 12 h (**A**) and the lysosomal inhibitor Baf A1 (200 nM) for 24 h (**B**) after *KDM5C* was knocked down using si*RNA*. Immunoblotting analysis was performed to assess the protein levels of CRBN and KDM5C.

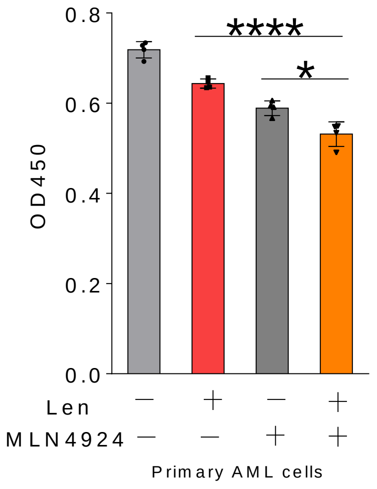

Supplemental Figure 5. MLN4924 enhances the antileukemia effect of Len. The primary AML cells were treated with MLN4924 (0.1 μM) for 18 h, and subsequently treated with lenalidomide (10 μM) for 4 days. Cell viability was measured by a CCK-8 assay. The data are shown as the means±SEMs (n = 4); *: $P < 0.05$; ****: $P < 0.0001$.

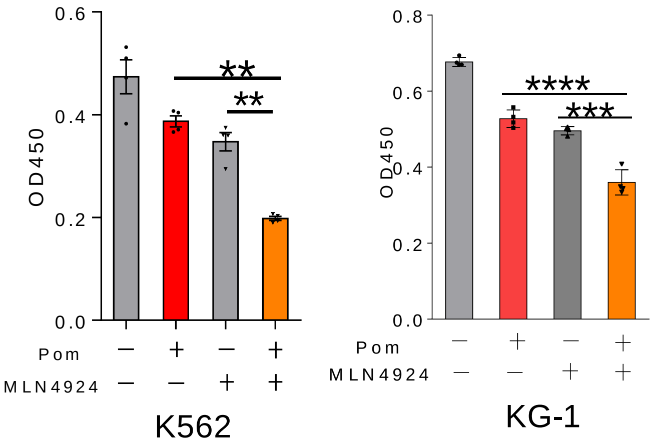

Supplemental Figure 6. MLN4924 enhances the antileukemia effect of Pom. K562 cells and KG-1 were treated with MLN4924 (40 nM) for 18 h, and subsequently treated with pomalidomide (2 μM) for 3 days (K562 cells) or 4 days (KG-1 cells). Cell viability was measured by a CCK-8 assay. The data are shown as the means±SEMs (n = 4), **: *P* < 0.01.

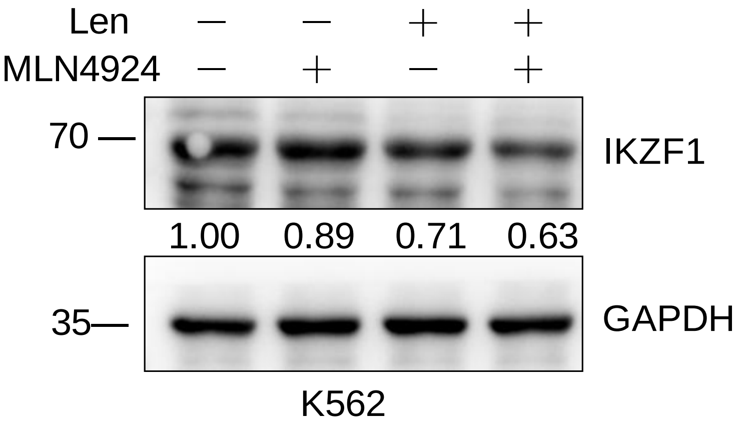

Supplemental Figure 7. MLN4924 could further decrease the Len-induced reduction of IKZF1. K562 cells were treated with MLN4924 (40 nM) for 18 h and then treated with lenalidomide (10 μM) for another 6 h. The cells were collected, lysed, and subjected to Western blotting analysis.
